# Association between Visceral and Bone Marrow Adipose Tissue and Bone Quality in Sedentary and Physically Active Ovariectomized Wistar Rats

**DOI:** 10.3390/life11060478

**Published:** 2021-05-25

**Authors:** Hélder Fonseca, Andrea Bezerra, Ana Coelho, José Alberto Duarte

**Affiliations:** 1 Faculty of Sport, University of Porto (FADE/UP), 4200-450 Porto, Portugal; up202000234@edu.fade.up.pt (A.B.); up201703666@edu.fade.up.pt (A.C.); jarduarte@fade.up.pt (J.A.D.); 2 Research Center of Physical Activity, Health and Leisure (CIAFEL), 4200-450 Porto, Portugal; 3 Laboratory for Integrative and Translational Research in Population Health (ITR), 4050-600 Porto, Portugal; 4 Polytechnic and University Higher Education Cooperative (CESPU), 4485-116 Gandra Campus, 4050-600 Porto, Portugal

**Keywords:** ovariectomy, bone quality, marrow adiposity, visceral adiposity, physical activity, running wheel exercise

## Abstract

Background: Obesity is considered protective for bone mass, but this view has been progressively challenged. Menopause is characterized by low bone mass and increased adiposity. Our aim was to determine how visceral and bone marrow adiposity change following ovariectomy (OVX), how they correlate with bone quality and if they are influenced by physical activity. Methods: Five-month-old Wistar rats were OVX or sham-operated and maintained in sedentary or physically active conditions for 9 months. Visceral and bone marrow adiposity as well as bone turnover, femur bone quality and biomechanical properties were assessed. Results: OVX resulted in higher weight, visceral and bone marrow adiposity. Visceral adiposity correlated inversely with femur Ct.Th (r = −0.63, *p* < 0.001), BV/TV (r = −0.67, *p* < 0.001), Tb.N (r = −0.69, *p* < 0.001) and positively with Tb.Sp (r = 0.58, *p* < 0.001). Bone marrow adiposity also correlated with bone resorption (r = 0.47, *p* < 0.01), bone formation rate (r = −0.63, *p* < 0.01), BV/TV (r = −0.85, *p* < 0.001), Ct.Th (r = −0.51, *p* < 0.0.01), and with higher empty osteocyte lacunae (r = 0.39, *p* < 0.05), higher percentage of osteocytes with oxidative stress (r = 0.64, *p* < 0.0.01) and lower femur maximal stress (r = −0.58, *p* < 0.001). Physical activity correlated inversely with both visceral (r = −0.74, *p* < 0.01) and bone marrow adiposity (r = −0.92, *p* < 0.001). Conclusions: OVX increases visceral and bone marrow adiposity which are associated with inferior bone quality and biomechanical properties. Physical activity could contribute to reduce adipose tissue and thereby improve bone quality.

## 1. Introduction

Obesity is one the most prevalent diseases worldwide [1] and a major risk factor for several health conditions [2] but also considered protective regarding bone mass [3] and fracture risk [4]. For instance, adolescents with higher adiposity have higher bone mass [5] while weight reduction leads to bone mass loss [6,7] and unfavorable geometry adaptations in the elderly [6]. Improvements in trabecular bone microarchitecture are also observed in animal models feed with high-fat diets [8]. Several mechanisms could explain these beneficial observations [9,10,11,12]. Nevertheless, this protective view of obesity over bone mass has been progressively challenged. This effect seems to be site dependent, more evident in normal and underweight persons, and has a lower impact on the obese [4], with obesity being associated with a reduction in fracture risk at central skeletal sites but with increases at peripheral sites [13,14]; this could be caused by adipose tissue-associated increases in cortical porosity [15]. Moreover, central adiposity seems to be particularly associated with increases in vertebral [16] and hip [17] fracture risk. The relation between bone and adipose tissue is therefore complex and poorly understood [18,19,20]. In addition, bone marrow adipose tissue, which could be expressed independently of other fat depots [21], also seems to negatively affect bone formation [22], bone mass [23,24] and to associate with lower trabecular bone mineral density (BMD) and higher vertebral fracture risk [25]. Nevertheless, the impact of marrow adiposity on bone quality is unclear because, although high marrow adiposity is present in many low-bone-mass disorders [21,26,27], this is not always the case [28]. Menopause is simultaneously characterized by rapid bone loss and weight gain [29]. Interestingly, adiposity in post-menopausal women seems to decrease the risk of major osteoporotic fractures [30] contrarily to men [31]. Estrogen loss following the menopause, a feature of normal ageing or surgical ovary removal, also favors bone marrow adiposity [32] which, mechanistically, could be associated with bone loss due to reduced osteoblast [33,34,35] and increased osteoclast differentiation [36], but preventing marrow adiposity expansion after the menopause has not been shown to prevent bone loss in experimental animals [37]. Exercise has well demonstrated beneficial effects on bone quality [38] and is a cornerstone in the treatment of postmenopausal osteoporosis [39]. Nevertheless, physical activity levels tend to decrease with ageing and, particularly in animal models, following estrogen loss. Although much of its effects result from direct bone tissue mechanical strain, considering that exercise also influences adipose tissue, namely at the bone marrow [40,41], it is undetermined if this could also significantly contribute to improvements in bone quality. Considering the complex relationship between adipose tissue and bone quality and that menopause is associated with adipose tissue expansion, the main purpose of this manuscript is to investigate how visceral and bone marrow adipose tissue associate with bone quality following estrogen loss and, to what extent, physical activity influences visceral and bone marrow adipose tissue and how that reflects on bone quality.

## 2. Materials and Methods

### 2.1. Experimental Design

Five-month-old nulliparous female Wistar rats (Charles River Laboratories, Barcelona, Spain) were either killed as baseline controls (BSL, n = 7) ovariectomized (OVX, n = 13) or sham operated (SHAM, n = 12). Bilateral OVX was performed by standard ventral approach under anesthesia with 4% sevoflurane in 2 L·min^−1^ O_2_. SHAM surgery was performed by exposing the ovaries without removal. OVX and SHAM animals were then allocated to a physically active (OVXEX, n = 7; SHAMEX, n = 6) or sedentary (OVXSED, n = 6; SHAMSED, n = 6) group. Physically active animals were housed in cages with running wheel and distance counter (Tecniplast, Buguggiate, Italy) allowing the animals to freely exercise at their will and to monitor their physical activity. The distance in the running wheel was recorded weekly throughout the experiment. Sedentary animals were housed in similar cages without running wheel. All animals were housed individually in an inverted 12/12 h light/dark cycle with access to food (Scientific Animal Food & Engineering; A04) and water ad libitum for 9 months. Body weight (BW) was determined weekly and BW gain calculated as current BW minus baseline BW.

### 2.2. Animals Sacrifice and Tissues Harvest

At 14 months of age, OVX and SHAM animals were anesthetized with 4% sevoflurane and killed. Blood samples were collected from the inferior vena cava, immediately centrifuged for 10 min, 500 g at 4 °C and the serum separated and stored at −80 °C for bone turnover markers and estrogen determination. Parametrial, retroperitoneal, inguinal and mesenteric fat depots were dissected and weighed (Kern 870; resolution 0.01 mg) for determining visceral fat. Both femora were removed and cleared of soft tissue. The right femur was saved in a sealed plastic bag at −80 °C wrapped in saline soaked gauze for biomechanical testing and dynamic histomorphometry. Left femur was fixed in 4% (*w*/*v*) paraformaldehyde (Sigma, St. Louis, MO, USA, 158127), 2.5% (*w*/*v*) sucrose (Sigma, S9378), 0.1% (*v*/*v*) glutaraldehyde (Sigma, G5882) and 10% (*w*/*v*) EDTA (Sigma, 03620) in phosphate buffer (PBS) pH 7.4 during 5 weeks at 4 °C for decalcification. Decalcifying endpoint was determined by the ammonium oxalate test [42].

### 2.3. Estradiol and Bone Turnover Markers

Ovariectomy was confirmed by determining serum 17β-estradiol concentration by ELISA (Demeditec Diagnostics, DE2693) as described previously [43]. All samples were analyzed in duplicate and standards in triplicate. Inter- and intra-assay coefficients of variation (CV) were under 9.4% and 6.8%, respectively. Assay range was 9.7–2000 pg·mL^−1^.

Bone formation was assayed by osteocalcin (OC) concentration with Rat-MID™ ELISA kit (Immunodiagnostic Systems, Boldon, UK). Assay range was between 50−1500 ng·mL^−1^. Inter and intra-assay CV were lower than 7.7% and 5.0%, respectively. Bone resorption was assayed by determining the concentration C-terminal telopeptides of type I collagen (CTX-I) with RatLaps™ EIA kit (AC-06F1, Immunodiagnostic Systems). Assay range was between 7.7–188 ng·mL^−1^. The inter and intra-assay CV were lower than 14.8% and 9.2%, respectively. Positive controls for OC and CTX-I were assayed together with samples and their concentration was found to be within the manufacturer’s quality control range.

### 2.4. Biomechanical Testing

Three-point bending of the right femur diaphysis was performed as described previously [44] according to the recommended guidelines [45,46] using a servo hydraulic testing machine (TIRATest 2705, TIRA GmbH, Schalkau, Germany). The femur was thawed overnight at 4 °C, its midpoint determined and positioned with posterior surface facing the machine supports who were then individually adjusted to be distal to the trochanter minor and proximal to the condyles. Average distance between the supports was 18.58 ± 0.59 mm (mean ± SD), corresponding to about 50% of femora length. A perpendicular, anterior–posterior load was then applied to the mid-diaphysis by a crosshead and load cell. A slow stabilizing preload (0.1 mm·s^−1^) was first applied until 5 N and afterwards rate increased to 0.5 mm·s^−1^ until failure. Bones were continually moistened in saline throughout the test. Cortical bone biomechanical properties were obtained from the computer generated load–deformation curve (TIRAsoft ZD1, TIRA GmbH, Germany) as detailed elsewhere [47]. Yield point was determined by the offset method using a 0.3% offset. The following bone tissue biomechanical parameters were computed: Young’s modulus (MPa), maximal stress (MPa), toughness to yield (MPa), post yield toughness (MPa), yield strain (%), yield stress (MPa), ultimate strain (%) and toughness (MPa). The distal femur specimen resulting from the three-point bending test was afterwards fixed in 4% (*w*/*v*) paraformaldehyde (Sigma 158127), 2.5% (*w*/*v*) sucrose (Sigma, S9378), 0.1% (*v*/*v*) glutaraldehyde (Sigma, G5882) in phosphate buffer (PBS) pH 7.4 for latter undecalcified dynamic histomorphometry analysis.

### 2.5. Tissue Preparation for Light Microscopy

Immediately after necropsy the left femur was fixed and decalcified as described above and processed for histological assessment of distal femur trabecular microarchitecture and mid-diaphysis cortical bone geometry, porosity, osteocyte density and bone marrow adiposity. The diaphysis midpoint was determined with a digital caliper and a 5 mm length bone section was separated with a scalpel from this region. Bone samples originating from the femur mid-diaphysis and distal extremity were dehydrated in graded ethanol (Panreac), cleared in xylene (Panreac), and imbedded in paraffin blocks (Merck). Serial sections (5 μm) from the mid-diaphysis (axial plane) and distal metaphysis intercondylar fossa (sagittal plane) were then obtained with a rotary microtome (Leica RM 2125). Sections were collected on silane-coated slides, dried, stained with Mayer’s hematoxylin (Sigma) and alcoholic eosin (Sigma) and mounted with DPX (Sigma) and glass coverslips.

### 2.6. Static Histomorphometry

Femur mid-diaphysis cortical bone geometry and distal metaphysis trabecular microarchitecture were determined according to recommendations published elsewhere [48]. Cortical bone sections stained with hematoxylin and eosin were viewed under a light microscope coupled to a digital camera (Axio Imager A1, Carl Zeiss) and analyzed with ImageJ (National Institutes of Health, USA). Cortical bone parameters measured were: cortical bone tissue area (Ct.Ar; mm^2^), cortical thickness (Ct.Th; μm) and marrow area (Ma.Ar; mm^2^). Diaphysis cross-sectional moment of inertia was also calculated from the histological sections of the left femur as described elsewhere [47] and used for calculation of right femur bone tissue biomechanical properties. Results were calculated as the average of five consecutive sections per animal.

Eight non-overlapping images, evenly distributed throughout each cortical bone section, each with 0.0910 mm^2^ area were used to determine osteocyte density (N.Ot/B.Ar; mm^2^) and empty osteocyte lacunae density (N.Lc/B.Ar; mm^2^) expressed as numbers per bone tissue area (B.Ar). Intracortical porosity area (Po.Ar) was determined on four images from the same cortical bone sections, as described previously [47]. Briefly, images were first converted to 16-bit color, followed by segmentation into bone tissue and pores using a fixed threshold. Each pore margin was then traced and its area determined. Pores with less than 25 μm^2^ (average size of osteocyte lacunae) were disconsidered. Pore area and cortical bone area of all images from the same histological section were then summed to give total pore and bone tissue area, respectively, and final results were expressed as the summed area of all pores per cortical bone tissue area (Po.Ar/Ct.Ar, %). Trabecular bone microarchitecture was determined in a 5.0 mm^2^ square region of the distal femur metaphysis at approximately 100 μm proximal to the growth plate. Trabecular microarchitecture parameters measured were: trabecular bone volume (BV/TV; %), trabecular thickness (Tb.Th; μm), trabecular number (Tb.N/mm^2^) and trabecular separation (Tb.Sp; μm). All parameters were determined as the average of five consecutive sections per animal.

### 2.7. Marrow Adiposity

Bone marrow adiposity (F.Ar/Ma.Ar; %) was assessed at the left femur mi-diaphysis cross section as the fraction of adipocytes area (F.Ar) relative to bone marrow area (Ma.Ar) assessed with ImageJ (National Institutes of Health, Bethesda, MD, USA). Marrow adiposity was determined in each animal as the average of 3 histological sections.

### 2.8. Dynamic Histomorphometry

Fifteen and 5 days prior to sacrifice all animals received 10 mg/Kg of calcein (Sigma, C0875) intraperitoneally in 0.9% saline with 2% (*w*/*v*) NaHCO_3_, pH 7.4. The right distal femur resulting from the 3-point bending test was used for dynamic histomorphometry after fixation with 4% paraformaldehyde. Approximately 3 mm thick sections of the diaphysis were obtained with a circular diamond saw 5 mm distal from the diaphysis failure point, dehydrated in graded alcohols, cleared in xylene and embedded undecalcified in methyl methacrylate (Sigma). Blocs were then mounted on glass slides, grounded to a final thickness of 20 µm, mounted with DPX and coverslips and analyzed under a fluorescent microscope coupled to a digital camera (Axio Imager A1, Carl Zeiss). Total, single, and double labeled surface and inter-label width were measured on periosteal and endosteal bone surfaces with ImageJ (National Institutes of Health, USA) for determination of mineralizing surface (MS/BS, %), mineral appositional rate (MAR, μm/d) and bone formation rate (BFR, µm^2^/µm/d) as defined elsewhere [49].

### 2.9. Detection and Quantification of Osteocytes with Oxidative Damage

Osteocytes positively stained for protein carbonyls by immunohistochemistry were quantified as a surrogate of bone tissue oxidative stress [50]. Briefly, after deparaffinization, cortical bone sections from de femur mid-diaphysis were washed in Tris-buffered saline (TBS, pH 7.2) and derivatized by incubation in 20 mM 2,4-dinitrophenylhydrazine (DNPH) in 10% (*v*/*v*) trifluoroacetic acid for 30 min at 25 °C. Negative controls were incubated with 2% (*v*/*v*) HCl solution. After blocking with 3% bovine serum albumin (BSA) in TBS for 30 min at 25 °C, sections were then incubated first with anti-DNPH-KLH antibody (Invitrogen A-6430) diluted 1:100 in TBS buffer for 1 h at 25 °C followed by incubation with goat anti-rabbit alkaline phosphatase conjugated antibody (Abcam ab6722) diluted 1:200 in TBS buffer for 1 h at 25 °C. Detection was performed by developing with Fast Red RC (Sigma F4648) followed by counterstaining with diluted hematoxylin. Total (Ot.N) and positively stained osteocytes (carbonyl N.Ot^+^) were quantified in eight equally distributed optical fields and results expressed relative to B.Ar.

### 2.10. Detection and Quantification of Osteocyte Apoptosis

Osteocyte apoptosis was assessed at the left femur mid-diaphysis decalcified cortical bone sections by terminal deoxynucleotidyl transferase dUTP nick end labeling (TUNEL) with a commercially available kit (in situ cell death detection kit AP, Roche) following the manufacturer instructions. After deparaffinization, antigen retrieval was performed by microwave irradiation at 750 W during 1 min. Sections were than blocked in 0.1 M Tris-HCl (pH 7.5) with 3%BSA for 30 min at 20 °C and afterwards incubated in TUNEL reaction solution for 60 min at 37 °C. Negative controls were prepared only with label solution. Sections were then incubated with converter AP for 30 min at 37 °C for allowing detection with Fast Red RC (Sigma F4648, Barcelona, Spain). The number of apoptotic osteocytes (TUNEL N.Ot^+^) was determined in eight equally distributed optical fields and results expressed relative to B.Ar.

### 2.11. Statistical Analysis

Data normality was assessed through the Kolmogorov–Smirnov test. Correlations between variables were assessed through Pearson correlation. For variables lacking normal distribution, bootstrapping with a sampling number of 1000 was applied. Cases were excluded pairwise. The effect of estrogen loss was assessed by comparing OVX and SHAM animals using either Mann–Whitney test or independent samples T test, whenever variables violated or not the assumptions for normal distribution, respectively. The effect of exercise was assessed using the same statistical approach, but comparing animals housed in physically active (EX) or sedentary (SED) conditions. Results were expressed as mean and standard deviation if not stated otherwise and 95% confidence intervals were calculated. Correlation coefficients and Cohen’s d were reported as effect size measures. Statistical tests were considered significant when *p* < *0*.05. Statistical analysis was performed with SPSS version 27.

## 3. Results

### 3.1. Effect of Ovariectomy on Body Weight, Adiposity and Bone Quality

**Figure 1:**
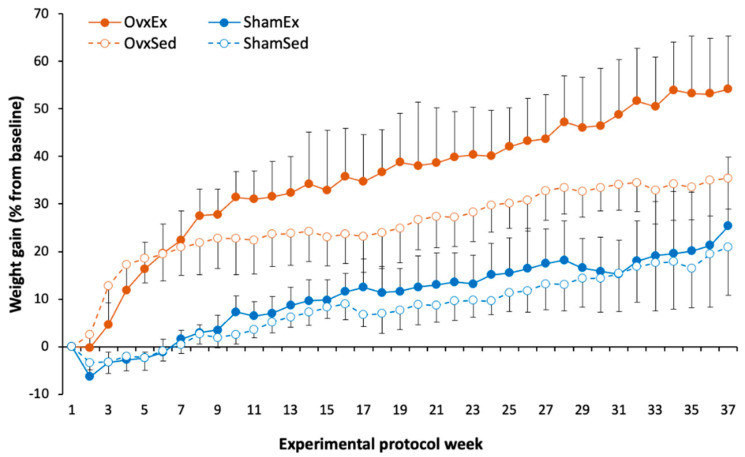
Average body weight gain percentage from baseline throughout the experimental period. Data points represented average body weight gain (%) in each group at each timepoint of the experiment and error bars the respective standard deviation.

**Figure 2:**
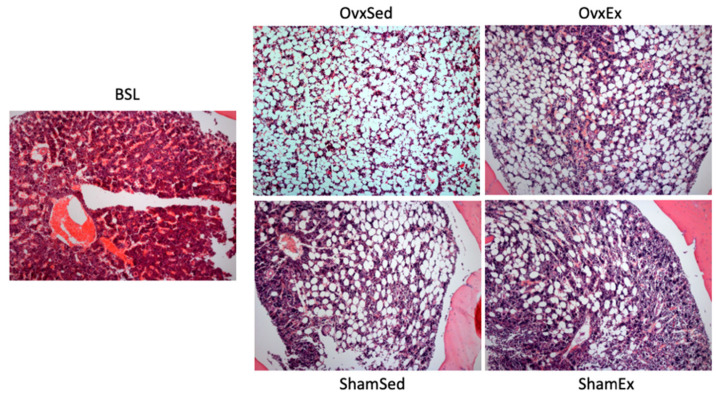
Bone marrow adiposity. Bone marrow adiposity was determined in histological sections of the decalcified left femur mid-diaphysis stained with hematoxylin and eosin. A higher number of bone marrow adipocytes can be appreciated in the upper panel corresponding to ovariectomized animals. BSL: baseline; Ovx: ovariectomy; Sham: sham operated; Ex: physically active; Sed: sedentary.

**Figure 3:**
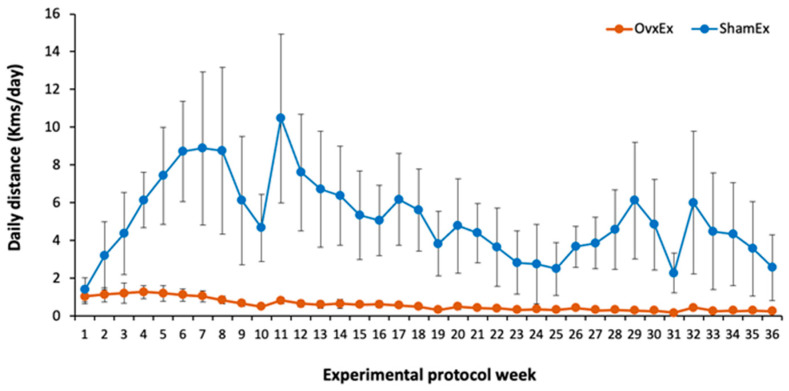
Running wheel activity. Data points represent the average daily distance of the animals in the running wheel during each one of the experimental protocol weeks while bars represent the respective standard deviation. A substantially lower running wheel activity in ovariectomized animals should be noted. Ovx: ovariectomy; Sham: sham operated; Ex: physically active; Sed: sedentary.

Successful ovariectomy was confirmed by the lower estradiol concentration on OVX compared to SHAM animals (18.4 ± 5.34 vs. 84.5 ± 17.40 pg/mL). At sacrifice OVX animals were heavier (Figure 1), had almost 2-fold higher intra-abdominal fat content (Table 1) and almost 3-fold higher bone marrow adiposity at the femur compared to SHAM animals (Figure 2). Since there were no significant differences between OVX and SHAM animals regarding food intake (Table 1), the higher body weight in OVX animals could be mostly explained by differences in energy expenditure, namely in locomotor activity, as OVX animals had significantly less running wheel activity compared to their SHAM counterparts (Figure 3).

**Figure 4:**
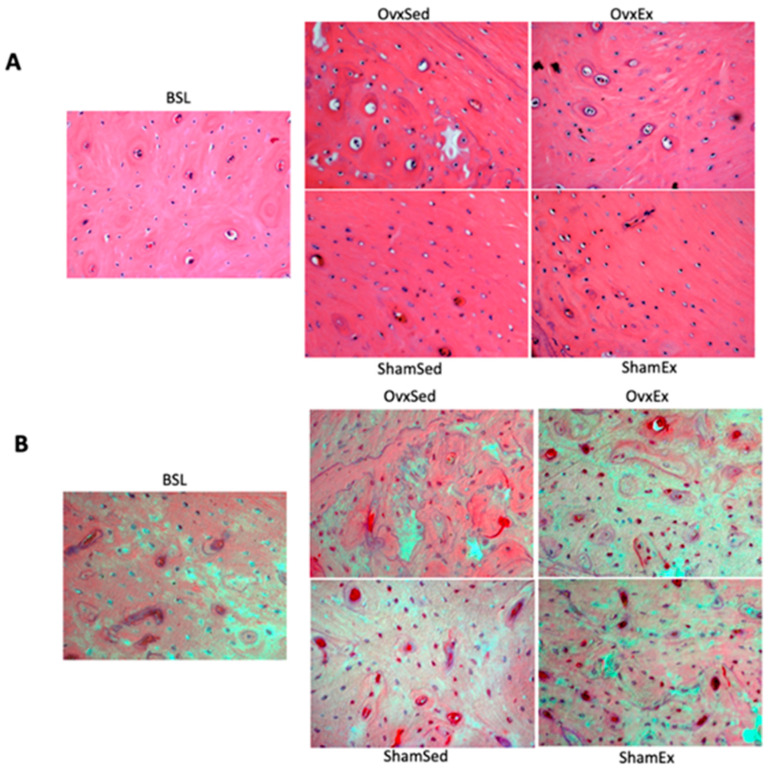
Osteocyte density and oxidative stress. Upper panel (**A**): Decalcified histological sections of the left mid-femur diaphysis stained with hematoxylin and eosin highlighting differences in osteocyte numbers per bone tissue area (osteocyte density) and the number of empty osteocyte lacunae. Lower panel (**B**): Decalcified histological sections of the left femur mid-diaphysis stained by immunohistochemistry for detection of protein carbonylation. Red color represents positive detection due to fast red formation. BSL: baseline; Ovx: ovariectomy; Sham: sham operated; Ex: physically active; Sed: sedentary.

Histological analysis to the femur mid-diaphysis showed that OVX animals had a significantly lower osteocyte density (Figure 4A) and higher number of osteocytes positively stained for protein carbonylation (Figure 4B), a sign of oxidative stress damage.

**Figure 5:**
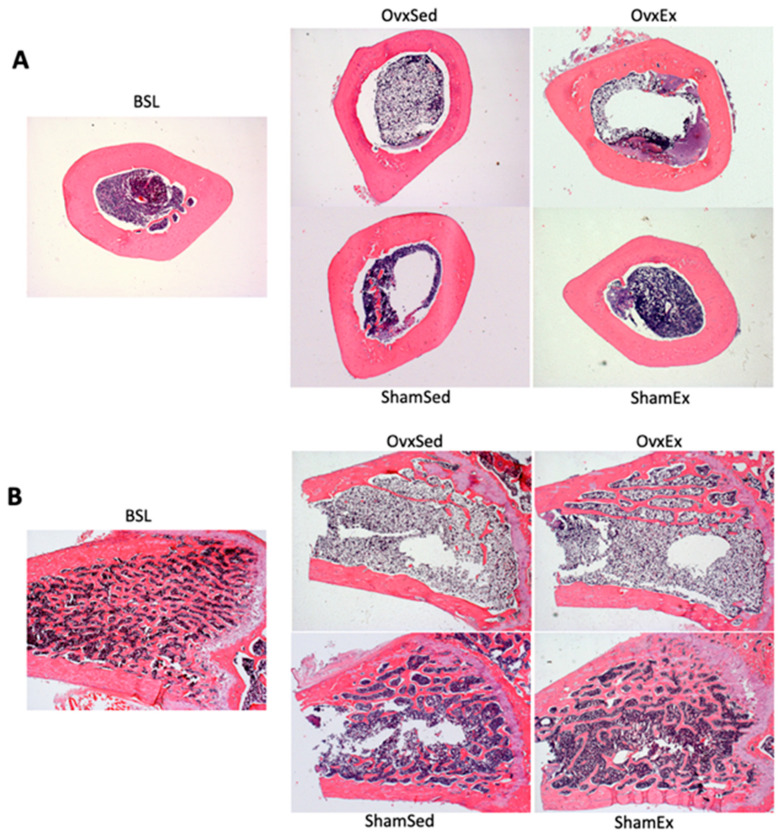
Cortical geometry and trabecular microarchitecture. Upper panel (**A**): Decalcified histological cross sections of the left femur mid-diaphysis stained with hematoxylin and eosin evidencing differences in cortical geometry between animals in the different groups. Lower panel (**B**): Decalcified histological sections (sagittal plane) of the left femur distal metaphysis stained with hematoxylin and eosin evidencing differences in trabecular microarchitecture between animals in the different groups. It is also possible to appreciate the different degree of marrow adiposity between animals with substantially higher marrow adiposity in OVX animals. BSL: baseline; Ovx: ovariectomy; Sham: sham operated; Ex: physically active; Sed: sedentary.

**Figure 6:**
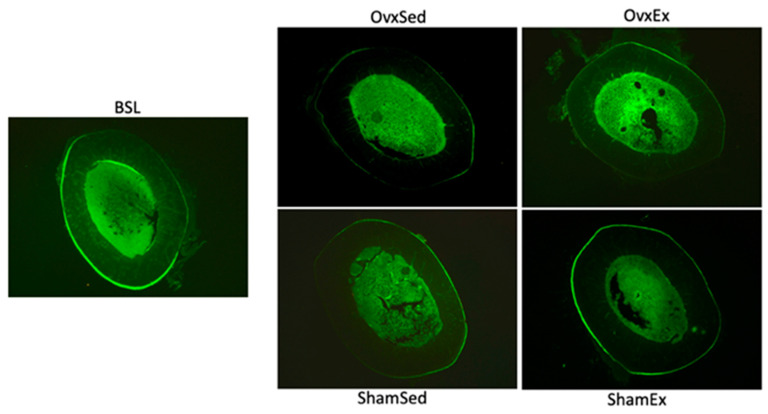
Dynamic histomorphometry. Undecalcified histological cross sections of the right femur mid-diaphysis viewed under fluorescent microscopy and labeled with calcein fluorochrome administered 15 and 5 days prior to the animals sacrifice. BSL: baseline; Ovx: ovariectomy; Sham: sham operated; Ex: physically active; Sed: sedentary.

Several bone quality parameters were also significantly inferior in OVX compared to SHAM animals, namely cortical thickness (Figure 5A), distal femur BV/TV and Tb.N were lower while Tb.Sp was higher in OVX animals (Figure 5B). Femur maximal stress during 3-point-bending was also significantly lower in OVX animals (Table 1). While there were no statistically significant differences in bone formation between OVX and SHAM animals both in terms of osteocalcin concentration and dynamic histomorphometry variables (Figure 6), bone resorption, assessed by CTX-I concentration, was higher in OVX animals compared to SHAM.

### 3.2. Association between Visceral Adipose Tissue and Bone Quality Markers

Higher body mass increases throughout the experiment (r = 0.64, CI: 0.33, 0.83; *p* < 0.001) and, in particular, a higher body mass at sacrifice (r = 0.89, CI: 0.77, 0.95; *p* < 0.001) correlated significantly with visceral adipose tissue accumulation. Visceral adipose tissue was also shown to correlate negatively with the average weekly distance traveled in the running wheel by the animals (r = −0.74, CI: −0.92, −0.32; *p* < 0.01) but not with the average weekly amount of food intake throughout the experiment (r = −0.016, CI: −0.41, 0.38; *p* = 0.94).

**Figure 7:**
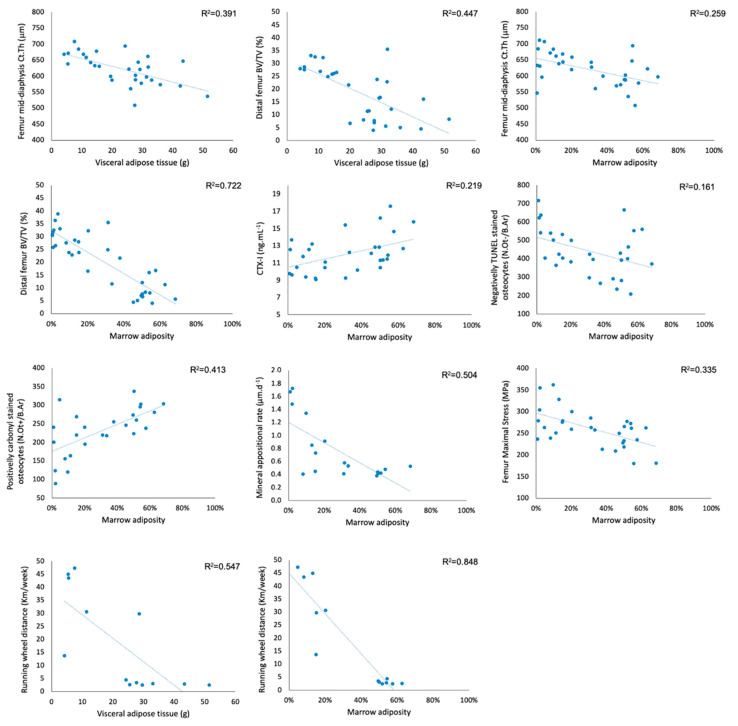
Correlations between visceral adipose tissue, bone marrow adiposity and several of the bone quality parameters assessed. Lower panels depict the significant negative correlation between both visceral and bone marrow adiposity and running wheel activity.

Regarding the association between visceral adipose tissue and bone quality (Figure 7), higher amounts of visceral adipose tissue were found to correlate negatively with femur diaphysis Ct.Th (r = −0.63, CI: −0.80, −0.34; *p* < 0.001), distal femur BV/TV (r = −0.67, CI: −0.83, −0.41; *p* < 0.001) and Tb.N (r = −0.69, CI: −0.84, −0.43; *p* < 0.001) and positively with Tb.Sp (r = 0.58, CI: 0.28, 0.78; *p* < 0.001). No significant correlations were identified between visceral adipose tissue amount and markers of bone turnover, femur biomechanical properties, osteocyte density, apoptosis or oxidative stress.

### 3.3. Association between Bone Marrow Adipose Tissue and Bone Quality Markers

Higher body mass increases throughout the experiment (r = 0.55, CI: 0.20, 0.78; *p* < 0.01), higher body mass at sacrifice (r = 0.77, CI: 0.59, 0.88; *p* < 0.001) and a higher amount of visceral adipose tissue (r = 0.65, CI: 0.37, 0.82; *p* < 0.001) all significantly correlated with a higher degree of bone marrow adiposity. Similarly to visceral adipose tissue, bone marrow adiposity seemed to be mostly associated with lower energy expenditure as it correlated strongly and inversely with running wheel activity (r = −0.92, CI: −0.98, −0.75; *p* < 0.001) but not with the average weekly amount of food intake (r = −0.22, CI: −0.56, 0.20; *p* = 0.301).

**Figure 8:**
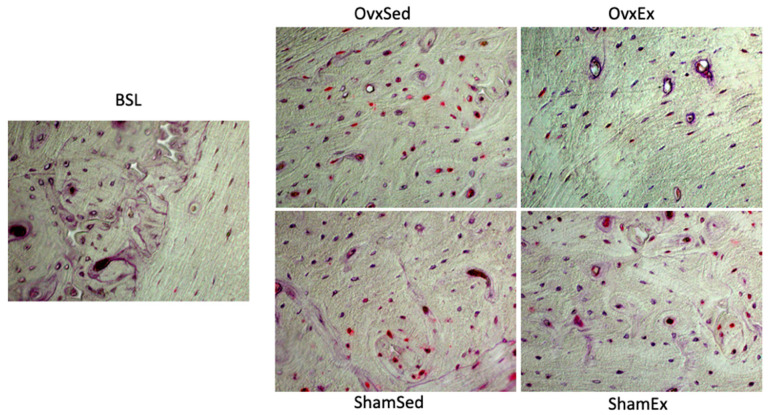
Osteocyte apoptosis. Decalcified histological sections of the left femur mid-diaphysis stained by the TUNEL method for detection of osteocyte apoptosis. Red color denotes positively (apoptotic) stained osteocytes. BSL: baseline; Ovx: ovariectomy; Sham: sham operated; Ex: physically active; Sed: sedentary.

Bone marrow adiposity was also found to have a significant inverse correlation (Figure 7) with femur mid-diaphysis Ct.Th (r = −0.51, CI: −0.73, −0.20; *p* < 0.01), BV/TV (r = −0.85, CI: −0.92, −0.72; *p* < 0.001) and Tb.N (r = −0.92, CI: −0.96, −0.84; *p* < 0.001) and to correlate positively with Tb.Sp (r = 0.77, CI: 0.58, 0.88; *p* < 0.001). Higher degrees of bone marrow adiposity were also found to negatively correlate with almost all femur diaphysis biomechanical properties assessed, namely with yield strain (r = −0.51, CI: −0.73, −0.18; *p* < 0.01), yield stress (r = −0.50, CI: −0.73, −0.17; *p* < 0.01), maximal stress (r = −0.58, CI: −0.78, −0.28; *p* < 0.001), toughness to yield (r = −0.55, CI: −0.76, −0.24; *p* < 0.01) and toughness (r = −0.39, CI: −0.66, −0.03; *p* < 0.05). The negative relationship between bone marrow adiposity and both cortical and trabecular microarchitecture and, consequently, bone biomechanical properties could be associated with either decreases in bone formation or increases in bone resorption since a significant positive correlation between bone marrow adiposity and CTX-I (r = 0.47, CI: 0.13, 0.71; *p* < 0.01) and an inverse correlation with MAR (r = −0.71, CI: −0.88, −0.36; *p* < 0.001) and BFR (r = −0.63, CI: −0.85, −0.23; *p* < 0.01) were also identified. Marrow adiposity was also found to correlate with the number of empty osteocyte lacunae (r = 0.39, CI: 0.05, 0.64; *p* < 0.05), the number of osteocytes displaying oxidate damage (r = 0.64, CI: 0.33, 0.83; *p* < 0.001) and to correlate inversely with the number of viable osteocytes negatively stained for TUNEL (Figure 8; r = −0.40, CI: −0.67, −0.04; *p* < 0.05).

### 3.4. Effect of Daily Physical Activity on Body Weight, Adiposity and Bone Quality

Although physically active animals had a higher food intake compared to sedentary counterparts there were no significant differences between groups regarding final body weight or body weight gain throughout the experiment (Table 2). There were also no differences between physically activity and sedentary animals regarding visceral adipose tissue or bone marrow adiposity. Irrespective of this, physically active animals displayed several improved parameters of bone quality such as higher femur cortical and trabecular bone thickness and lower femur mid-diaphysis cortical porosity. Physically active animals also had higher osteocyte density and lower percentage of osteocytes positively stained for TUNEL. Femur maximal stress was also higher in physically active animals compared to sedentary controls.

## 4. Discussion

The main purposes of this study were to determine how OVX affected visceral and bone marrow adipose tissue, how these fat depots correlated with bone quality traits and to determine if physical activity affected both visceral and bone marrow adiposity and, if this was the case, how this reflected on bone quality in OVX animals. Our findings show that OVX resulted in a significant increase in body weight and in a significant expansion of both visceral and bone marrow adipose tissue depots. Visceral and, in particular, bone marrow adipose tissue were both shown to correlate with several indicators of bone quality deterioration as well as to be inversely associated with femur biomechanical properties. Physical activity in turn, determined by the running distance on the activity wheel, correlated strongly and inversely with visceral and, particularly, with bone marrow adiposity suggesting that physical activity could have the potential to improve bone quality independently of mechanical strain but also as a result of its favorable metabolic effects on adipose tissue.

### 4.1. OVX Leads to Body Weight Gain, Visceral and Bone Marrow Adipose Tissue Increase

A redistribution of adipose tissue from peripheral to central depots following estrogen loss, resulting in increases in visceral [51] and bone marrow adiposity [32] have been described previously. Post-menopausal women were shown to have a higher amount of visceral adipose tissue compared to pre-menopausal women [52,53] while a 10% increase in visceral fat has been described in female Wistar rats following just 2 months following OVX [54]. Additionally, OVX animals treated with estradiol for 4 weeks gained less weight and less visceral adipose tissue compared to non-treated controls [55]. The expansion of visceral adipose tissue following estrogen loss could result from several mechanisms, namely lower lipolysis and increased adipocyte lipoprotein lipase activity resulting in increased adipocyte size [52]. With this peripheral to central fat distribution somewhat resembling a Cushingoid phenotype, it is also not surprising that one of the mechanisms explaining visceral adipose tissue increase following estrogen loss is associated with local visceral adipose tissue changes in glucocorticoid metabolism [55] even though cortisol levels in postmenopausal women tend to remain unaltered [56]. Why estrogen loss affects more profoundly some adipose tissue depots than others might result from differences in estrogen receptor isoform expression [57].

Our results showing a consistent increase in bone marrow adiposity following estrogen loss are also in line with findings from previous studies both in humans [58] and in animal models [59]. In addition, there is also evidence that treatment with estrogen results in a decrease in bone marrow adiposity, a finding that has also been replicated in humans [32] and experimental animals [59]. The effects of estrogen on marrow adiposity are observed not just after long-term loss but also in the short term, since marrow adipose tissue has been shown to vary during the menstrual cycle [60] and, in post-menopausal women, to decrease after short-term treatment with estrogen and increase again after treatment cessation [60]. Several mechanisms have been suggested for the estrogen deficiency associated marrow adipose tissue expansion. Namely, estrogen has been shown to suppresses bone marrow adipocyte differentiation through transforming growth beta (TGF-β) mediated induction of connective tissue growth factor [61], through estrogen receptor alfa (ERα) signaling [62], sclerostin inhibition [63,64], as well as by suppressing proinflammatory cytokines [65,66] and oxidative stress [67].

### 4.2. Association between Visceral and Bone Marrow Adiposity and Bone Quality

Collectively, our results showed that there is a strong inverse relationship between excess adiposity and bone quality which therefore suggests that adipose tissue expansion following estrogen loss could possibly be one of the mechanisms leading to reduced bone quality and favoring increases in bone fragility. In our study, animals that gained more weight also had more visceral adipose tissue and this increase, in turn, was associated with a reduction in femur mid-diaphysis cortical thickness and distal femur trabecular microarchitecture deterioration. Previous studies have shown that central adiposity is associated with increased risk of both vertebral [16] and hip [17] fractures. In a large sample of females between 45–70 years, a significant inverse association between visceral adipose tissue and bone mineral density after adjusting for body mass and lifestyle factors was found [68]. This inverse association between visceral adipose tissue and bone mass was mostly apparent in those with overweight. These results are consistent with our findings and with the notion that high amounts of visceral adipose tissue may be deleterious to bone metabolism. Although our experimental protocol does not enable us to determine if the observed relation is merely an association or if there is causal relationship, several mechanisms support the plausibility of a causal relationship. Higher amounts of visceral adipose tissue are associated with increases in circulating proinflammatory cytokines [69,70] which favors osteoclast differentiation and survival and bone resorption. Irrespective of this, in our study we did not identified any significant correlation between visceral adipose tissue and bone resorption markers. Additionally, higher amounts of visceral adipose tissue have been shown to negatively affect insulin-like growth factor metabolism [71] which, in turn, could negatively affect bone formation. Once more, however, our results do not support this since we identified no significant correlation between visceral adipose tissue and either biochemical or histological markers of bone formation.

Our results also showed that there is a strong association between higher marrow adiposity and reduced bone quality. While visceral adipose tissue only correlated with cortical and trabecular deterioration, higher marrow adiposity was show to correlate negatively with cortical geometry, trabecular microarchitecture, osteocyte death and bone turnover. Most importantly, a higher degree of marrow adiposity was associated with significantly lower femur resistance to fracture with almost all of the femur mid-diaphysis cortical bone biomechanical properties being negatively associated with marrow adiposity.

Osteoblasts and adipocytes belong both to a mesenchymal cell lineage and, therefore, share the same undifferentiated precursor cell [72]. Therefore, the number of osteoblasts and marrow adipocytes necessarily result from the commitment of the mesenchymal progenitor towards one lineage or the other which consequently results in that increases in one lineage necessarily occur at the expense of the other [73]. Cell culture studies using stromal progenitor cells clearly show that estrogen increases the expression of osteoblast differentiation markers and reduces lipid accumulation which is reversed with an estrogen antagonist [34] while in post-menopausal women, treatment with estrogen decreases vertebral bone marrow adiposity while concomitantly increases bone formation and decreases bone resorption [60]. Because of this, accumulating evidence suggests that abnormal expansion of marrow adipose tissue following estrogen loss after OVX or menopause plays a crucial role in bone loss. Our results are in line with this view as we showed a significant inverse correlation between marrow adiposity and dynamic histomorphometry markers of bone formation, namely with MAR and BFR.

In addition to the fact that expansion of marrow adipose tissue could happen at the expense of the reduction in available osteoblasts and, consequently of bone formation, there is also evidence that bone marrow adipocytes exert a local lipotoxicity effect, secreting free fatty acids that, through local paracrine signaling favor PPAR-γ and reduce Runx2 activity thereby reducing osteoblast differentiation, proliferation and activity [34,74,75]. One of the mechanisms through which adipocyte secreted factors have been suggested to negatively affect osteoblasts is also through increases in oxidative stress [74]. In addition, high levels of oxidative stress accompanying estrogen loss can also inhibit osteoblast differentiation and promote adipocyte lineage drift from mesenchymal precursors [67], thereby also contributing to reduced bone formation in a feed-forward mechanism. Our results are in line with these findings as we showed significantly higher number of osteocytes displaying oxidative stress damage in OVX animals as well as a significant correlation between osteocytes positively stained for protein carbonylation, a marker of oxidative stress, and bone marrow adiposity.

Both our results and findings from others also suggest that marrow adiposity is not only associated with reduced bone formation but also with increased bone resorption. Our results showed a strong correlation between bone resorption marker CTX-I and marrow adiposity. This increased bone resorption could result from a direct effect of marrow adipocytes supporting osteoclast differentiation and activity. For instance, Beekman et al. [76] showed that OVX significantly increases the percentage of RANKL bone marrow expressing adipocytes compared to SHAM operated controls. More recently it was shown that increased bone resorption in OVX mice was partially attenuated by suppressing RANKL expression on bone marrow adipogenic precursors [77]. PPAR-γ mediated activation of c-fos has also been shown to mediate osteoclast differentiation independently of RANKL [78] which could also mechanistically explain the association between marrow adiposity and increased bone resorption in our results.

Lipid accumulation in several tissues also induces mitochondrial DNA damage and mitochondrial dysfunction leading thereby to increases in oxidative stress, protein degradation and apoptosis [79]. Independently of oxidative stress, marrow adiposity can also induce apoptosis in osteocytes by the direct action of palmitic acid, an abundant fatty acid in the bone marrow, particularly when there is increased bone marrow adiposity [80]. These mechanisms also support our findings of a significant correlation between marrow adiposity and higher numbers of positively stained osteocytes for protein carbonylation, positively TUNEL stained osteocytes and with empty osteocyte lacunae numbers, which are all indicators for higher osteocyte oxidative stress damage, apoptosis and death, respectively.

Therefore, both our results and several other findings suggest that estrogen loss associated adipose tissue expansion, in particular at the bone marrow, both affects bone formation and resorption, unbalancing bone turnover and leading thereby to bone loss. Our findings clearly support this relationship with the identification of an inverse correlation between marrow adiposity and parameters of both cortical and trabecular bone quantity ultimately compromising bone biomechanical properties.

### 4.3. Effects of Physical Activity on Visceral and Bone Marrow Adiposity

One of the central purposes of this study was to investigate if physical activity significantly influenced the degree of adiposity in OVX animals and if this, thereby, also influenced bone quality. Our results show that both visceral adipose tissue and, in particular, bone marrow adiposity were significantly and inversely correlated with the animals average weekly running wheel activity. In light of the findings discussed above and the mechanisms supporting the negative effect that both visceral and bone marrow adipose tissue have on bone metabolism, these correlations support the notion that higher levels of physical activity likely also have a protective effect on bone metabolism through modulation of adipose tissue depots.

Previous studies on experimental animals using forced exercise training models also show that exercise training is able to reduce visceral and bone marrow adiposity. For instance, a significant reduction in visceral adipose tissue was identified after 10-weeks of resistance training in OVX animals [81]. In addition to exercise training, voluntary running wheel activity for 6 weeks also resulted in a significant decrease in marrow adipose tissue expansion in mice treated with the PPAR-γ agonist rosiglitazone which is associated with both bone marrow expansion and increased fracture risk [41]. In experimental animals fed a high-fat diet, voluntary running wheel activity also limited bone marrow adipose tissue expansion and significantly improved trabecular microarchitecture [40]. Reduced marrow adiposity in physically active high-fat fed mice has been suggested to result from increased exercise induced adipose tissue β-oxidation [82].

Our results showed that OVX had a profound influence on the animals weight gain and both visceral and bone marrow adipose tissue expression. Since food intake was similar between OVX and SHAM animals, these differences most likely resulted from the substantially lower locomotor activity observed in OVX animals. Very similar findings were described by Rogers et al. [83] in which OVX mice that were monitored through a 12-weeks period gained 25% more weight and about 4.5 fold higher visceral adipose tissue compared to SHAM animals. Additionally, in Rogers et al.’s study [83], differences were attributable not to differences in food intake, but to differences in locomotor activity. These findings support our results and highlight that much of the negative effects of estrogen loss on adipose tissue expression and, consequently, on bone metabolism, could result from decreases in voluntary motor activity. In fact, our results showed that OVX had a profound influence on the animals running wheel activity. This effect of estrogen loss has already been robustly demonstrated by several experiments on animal models [84,85], but if this is also the case in humans and, therefore, if a reduction in daily physical activity also contributes to adipose tissue expansion and thereby to dysregulated bone metabolism in humans following the menopause remains to be determined.

Despite the negative correlations observed between both visceral and bone marrow adipose tissue and running wheel activity, no significant differences were observed between sedentary and physically active animals for both visceral and bone marrow adiposity. This finding suggests that, the volume of running wheel activity was insufficient to result in a significant decrease in bone marrow adiposity which again, could result from the negative impact that estrogen loss had on the animal’s locomotor activity.

## 5. Conclusions

In conclusion, our results suggest that OVX leads to a significant body weight gain over time which, in turn, is accompanied by increases in visceral and bone marrow adipose tissue. Higher visceral adipose tissue and, most importantly, higher bone marrow adiposity are associated with substantial bone quality deterioration and, in the case of marrow adiposity, with reduced bone biomechanical properties. Physical activity in turn, is inversely correlated with both visceral and bone marrow adipose tissue gain. Nevertheless, since OVX leads to a substantial decrease in locomotor activity, voluntary running wheel activity volume was likely insufficient to significantly decrease visceral and bone marrow adiposity in physically active animals in comparison to sedentary controls.

## Figures and Tables

**Table 1:** Comparisons between ovariectomized (OVX) and sham operated (SHAM) animals.

	OVX			SHAM							
	N	Mean	SD	N	Mean	SD	*p*	Mean dif.	95% CI		Cohen *d*
Food intake (g/week)	13	132.4	18.6	12	134.2	22.0	0.831	−1.8	−18.5	15.0	−0.09
Running wheel (Km/w)	7	2.98	0.69	6	34.95	12.83	**<0.001**	−33.69	−42.51	−25.36	−3.69
Initial BW (g)	13	263	23	12	256	20	0.378	7.7	−10.0	25.4	0.36
* BW at sacrifice (g)	13	379.3	37.7	12	312.1	38.7	**<0.001**	73.9	35.0	100.0	1.76
BW gain (g)	13	46.0	12.9	12	22.8	13.6	**<0.001**	23.2	12.2	34.1	1.75
Visceral fat (g)	13	32.4	8.9	12	17.9	11.2	**0.002**	14.5	6.2	22.8	1.44
* OC (ng.mL^−1^)	10	76.33	29.20	10	56.78	20.23	0.143	15.78	−6.06	41.04	0.78
CTX-I (ng.mL^−1^)	13	13.16	2.20	12	11.24	1.89	**0.028**	1.93	0.22	3.63	0.94
* Ct.Ar (mm^2^)	13	5.173	0.340	12	5.280	0.376	0.650	−0.083	−0.443	0.170	−0.30
Ct.Th (μm)	13	591.3	46.3	12	641.3	37.7	**0.007**	−50.0	−85.1	−14.8	−1.18
* Po.Ar/Ct.Ar (%)	13	1.369	1.098	12	1.175	0.725	0.936	0.020	−0.503	0.683	0.21
* F.Ar/Ma.Ar (%)	13	53.76	6.33	12	20.25	10.84	**<0.001**	34.98	24.61	42.01	3.82
BV/TV (%)	13	8.69	4.18	12	25.48	6.89	**<0.001**	−16.79	−21.46	-12.12	−2.98
Tb.Th (μm)	13	63.01	7.92	12	60.50	6.17	0.389	2.51	−3.40	8.42	0.35
*Tb.N (/mm^2^)	13	1.344	0.515	12	4.154	0.846	**<0.001**	−2.88	−3.43	−2.30	−4.05
*Tb.Sp (μm)	13	786.8	323.8	12	192.3	71.7	**<0.001**	559.4	367.2	838.0	2.49
N.Ot/B.Ar (/mm^2^)	13	605	50	12	648	47	**0.040**	−42	−83	−2	−0.87
N.Lc/B.Ar (/mm^2^)	13	312	89	12	318	63	0.858	−6	−69	58	−0.07
* Oc.N/Lc.N	13	2.209	1.140	12	2.143	0.608	0.574	−0.115	−0.570	0.410	0.07
TUNEL N.Ot+/B.Ar (/mm^2^)	12	261	176	12	294	105	0.587	−33	−155	90	−0.22
TUNEL N.Ot−/B.Ar (/mm^2^)	12	404	140	12	411	83	0.886	−7	−104	91	−0.06
TUNEL N.Ot+/N.Ot total (%)	12	38	20	12	41	13	0.676	−3	−18	12	−0.17
Carbonyl N.Ot+/B.Ar (/mm^2^)	10	276	35	10	225	48	**0.015**	51	11	91	1.21
Carbonyl N.Ot−/B.Ar (/mm^2^)	11	333	75	11	453	96	**0.004**	−121	−198	−44	−1.40
Carbonyl N.Ot+/N.Ot total (%)	11	45	8	11	34	9	**0.008**	10	3	18	1.27
MS/BS (%)	6	43.1	11.5	8	51.9	12.1	0.198	−8.8	−22.8	5.2	−0.74
* MAR (μm.d^−1^)	6	0.442	0.053	8	0.606	0.200	0.142	−0.115	−0.413	0.017	−1.05
* BFR (µm2/µm/d)	6	1.097	0.390	8	1.167	0.254	0.491	−0.143	−0.420	0.284	−0.22
Young´s Modulus (MPa)	13	15185	3040	12	16900	2602	0.145	−1715	−4066	636	−0.60
Maximal Stress (MPa)	13	236.2	32.6	12	267.6	29.4	**0.019**	−31.4	−57.2	−5.7	−1.01
Toughness to Yield (MPa)	13	1.327	0.376	12	1.517	0.295	0.177	−0.189	−0.471	0.092	−0.56
Postyield toughness (MPa)	13	2.041	0.532	12	2.094	0.955	0.863	−0.053	−0.686	0.580	−0.07
* Yield Strain (%)	13	1.31	0.025	12	1.36	0.12	0.225	−0.06	−0.22	0.11	−0.29
* Yield Stress (MPa)	13	185.1	32.8	12	209.8	34.6	0.123	−18.5	−50.8	3.9	−0.73
* Ultimate strain (%)	13	2.27	0.39	12	2.24	0.35	1.000	0.03	−0.28	0.34	0.09
* Toughness (MJ)	13	3.368	0.700	12	3.611	0.790	0.470	−0.201	−0.801	0.402	−0.33

Body weight (BW) gain was determined as the difference between BW at sacrifice and initial BW. Comparisons were performed with Independent samples T test except for variables marked with an * in which comparisons were performed with the Mann–Whitney test due to lack of the necessary assumptions for parametric testing. OC: Osteocalcin; CTX-I: carboxy-terminal collagen crosslinks of collagen type I; Ct.Ar: cortical bone tissue area; Ct.Th: cortical thickness; Po.Ar/Ct.Ar: cortical porosity; F.Ar/Ma.Ar: marrow adiposity; BV/TV: trabecular bone volume; Tb.Th: trabecular thickness; Tb.N: trabecular number; Tb.Sp: trabecular separation; N.Ot/B.Ar: osteocyte number/bone tissue area; N.Lc/B.Ar: empty osteocyte lacunae/bone tissue area; Oc.N/Lc.N: osteocyte/empty lacunae ratio; TUNEL N.Ot+/B.Ar: Terminal deoxynucleotidyl transferase dUTP nick end labeling (TUNEL) positive osteocytes/bone tissue area; TUNEL N.Ot−/B.Ar: TUNEL negative osteocytes/bone tissue area; TUNEL N.Ot+/N.Ot total: TUNEL+ osteocytes/total osteocyte number; Carbonyl N.Ot+/B.Ar: carbonyl group positive osteocytes/bone tissue area; Carbonyl N.Ot−/B.Ar: carbonyl group negative osteocytes/bone tissue area; Carbonyl N.Ot+/N.Ot total: carbonyl group positive osteocytes/total.

**Table 2:** Comparisons between sedentary and physically active animals.

	Sedentary			Physically Active							
	N	Mean	SD	N	Mean	SD	*p*	Mean dif.	95% CI		Cohen *d*
Food intake (g/week)	12	116.1	8.6	13	149.2	12.3	**<0.001**	−33.1	−41.9	−24.2	−3.09
Initial BW (g)	12	268.5	22.4	13	251.8	17.3	**0.048**	16.7	0.2	33.2	0.84
* BW at sacrifice (g)	12	343.6	40.2	13	350.2	60.3	0.852	−6.6	−49.4	36.3	−0.13
BW gain (g)	12	28.4	12.5	13	40.8	19.8	0.075	−12.5	−26.3	1.4	−0.75
Visceral fat (g)	12	28.1	7.9	13	23.0	15.2	0.299	5.1	−4.9	15.2	0.42
* OC (ng.mL^−1^)	9	73.7	32.9	11	60.7	19.4	0.370	13.0	−11.8	37.8	0.50
CTX-I (ng.mL^−1^)	12	12.92	2.72	13	11.61	1.54	0.161	1.31	−0.58	3.19	0.60
* Ct.Ar (mm^2^)	12	5.173	0.385	13	5.272	0.332	0.538	−0.099	−0.395	0.198	−0.28
Ct.Th (μm)	12	595.4	40.8	13	633.6	49.6	**0.048**	−38.2	−76.0	−0.4	−0.84
* Po.Ar/Ct.Ar (%)	12	1.90	0.99	13	0.70	0.26	**<0.001**	1.20	0.56	1.84	1.69
* F.Ar/Ma.Ar (%)	12	40.3	15.8	13	35.2	22.1	0.810	5.1	−10.8	20.9	0.26
BV/TV (%)	12	13.84	10.27	13	19.43	9.73	0.176	−5.59	−13.86	2.69	−0.56
Tb.Th (μm)	12	58.17	6.60	13	65.16	5.96	0.011	−7.00	−12.19	−1.80	−1.12
* Tb.N (/mm^2^)	12	2.38	1.66	13	2.98	1.52	0.137	−0.61	−1.92	0.71	−0.38
* Tb.Sp (μm)	12	640	470	13	374	232	0.110	266	−53	585	0.73
N.Ot/B.Ar (/mm^2^)	12	598	50	13	651	42	**0.009**	−53	−90	−15	−1.15
N.Lc/B.Ar (/mm^2^)	12	370	40	13	264	65	**<0.001**	106	61	150	1.95
* Oc.N/Lc.N	12	1.64	0.22	13	2.68	1.02	**<0.001**	−1.04	−1.66	−0.42	−1.39
TUNEL N.Ot+/B.Ar (/mm^2^)	12	372	138	12	183	66	**<0.001**	188	97	280	1.74
TUNEL N.Ot-/B.Ar (/mm^2^)	12	325	71	12	489	83	**<0.001**	−164	−229	−98	−2.12
TUNEL N.Ot+/N.Ot total (%)	12	52.1	13.2	12	27.3	9.2	**<0.001**	24.8	15.2	34.5	2.18
Carbonil N.Ot+/B.Ar (/mm^2^)	9	245	51	11	255	49	0.674	−10	−57	37	−0.19
Carbonil N.Ot-/B.Ar (/mm^2^)	11	367	110	11	419	97	0.257	−52	−144	41	−0.50
Carbonil N.Ot+/N.Ot total (%)	11	40.8	10.2	11	38.4	9.5	0.572	2.4	−6.3	11.2	0.25
MS/BS (%)	6	48.5	13.0	8	47.8	12.6	0.925	0.7	−14.4	15.7	0.05
* MAR (μm.d^−1^)	6	0.482	0.071	8	0.576	0.217	0.852	−0.094	−0.280	0.093	−0.54
* BFR (µm2/µm/d)	6	1.022	0.147	8	1.223	0.374	0.414	−0.201	−0.555	0.154	−0.67
Young Modulus (MPa)	12	15030	3205	13	16912	2392	0.108	−1882	−4209	445	−0.67
Maximal Stress (MPa)	12	235.9	34.6	13	265.5	28.5	**0.028**	−29.6	−55.7	−3.4	−0.94
Toughness to Yield (MPa)	12	1.296	0.360	13	1.532	0.303	0.088	−0.236	−0.511	0.038	−0.71
Postyield toughness (MPa)	12	2.127	0.900	13	2.011	0.609	0.708	0.116	−0.515	0.747	0.15
* Yield Strain (%)	12	1.33	0.24	13	1.34	0.16	0.650	−0.02	−0.18	0.15	−0.08
* Yield Stress (MPa)	12	182.3	35.6	13	210.4	30.3	0.168	−28.1	−55.4	−0.9	−0.85
* Ultimate strain (%)	12	2.33	0.43	13	2.19	0.30	0.470	0.13	−0.17	0.44	0.37
* Toughness (MPa)	12	3.422	0.916	13	3.542	0.563	0.437	−0.120	−0.743	0.503	−0.16

Body weight (BW) gain was determined as the difference between BW at sacrifice and initial BW. Comparisons were performed with Independent samples T test except for variables marked with an * in which comparisons were performed with the Mann–Whitney test due to lack of the necessary assumptions for parametric testing. OC: Osteocalcin; CTX-I: carboxy-terminal collagen crosslinks of collagen type I; Ct.Ar: cortical bone tissue area; Ct.Th: cortical thickness; Po.Ar/Ct.Ar: cortical porosity; F.Ar/Ma.Ar: marrow adiposity; BV/TV: trabecular bone volume; Tb.Th: trabecular thickness; Tb.N: trabecular number; Tb.Sp: trabecular separation; N.Ot/B.Ar: osteocyte number/bone tissue area; N.Lc/B.Ar: empty osteocyte lacunae/bone tissue area; Oc.N/Lc.N: osteocyte/empty lacunae ratio; TUNEL N.Ot+/B.Ar: Terminal deoxynucleotidyl transferase dUTP nick end labeling (TUNEL) positive osteocytes/bone tissue area; TUNEL N.Ot-/B.Ar: TUNEL negative osteocytes/bone tissue area; TUNEL N.Ot+/N.Ot total: TUNEL+ osteocytes/total osteocyte number; Carbonyl N.Ot+/B.Ar: carbonyl group positive osteocytes/bone tissue area; Carbonyl N.Ot-/B.Ar: carbonyl group negative osteocytes/bone tissue area; Carbonyl N.Ot+/N.Ot total: carbonyl group positive osteocytes/total osteocyte number; MS/BS: Mineralizing surface/bone surface; MAR: mineral appositional rate; BFR: bone formation rate.
